# Platelet aggregation and clot formation in comatose survivors of cardiac arrest treated with induced hypothermia and dual platelet inhibition with aspirin and ticagrelor; a prospective observational study

**DOI:** 10.1186/s13054-014-0495-z

**Published:** 2014-09-30

**Authors:** Thomas Kander, Josef Dankiewicz, Hans Friberg, Ulf Schött

**Affiliations:** Department of Intensive and Perioperative Care, Skåne University Hospital and Lund University, Lund, Sweden

## Abstract

**Introduction:**

We conducted a prospective observational study in cardiac arrest survivors treated with mild induced hypothermia, evaluating different platelet function tests at hypo- and normothermia. We also investigated the relation between gastric emptying and vasodilator stimulated phosphoprotein (VASP).

**Methods:**

Comatose survivors of out of hospital cardiac arrest were included and divided into two groups, depending on whether dual platelet inhibition with peroral ticagrelor and aspirin was given or not. The first blood samples (T1) were collected 12–24 hours after reaching target temperature (33°C) and were compared to blood samples collected 12–28 hours after reaching normothermia (37°C) (T2) within each group. All samples were analysed by Sonoclot viscoelasticity, flow cytometry based VASP and with multiple electrode aggregometry, Multiplate®; adenosine diphosphate (ADP), collagen (COL), thrombin receptor agonist peptide (TRAP) and arachidonic acid (ASPI). Sonoclot and Multiplate® instruments were set on in vivo temperatures. Gastric secretion from the nasogastric tube was measured to assess absorption of per orally administered antiplatelet drugs. Differences between T1 and T2 within each group were calculated using Wilcoxon matched pairs signed test. Significance levels were set at *P* <0.01.

**Results:**

In total, 23 patients were included. In patients with dual platelet inhibition (n =14) Multiplate®-analyses showed no changes in ADP stimulated platelets. COL, TRAP and ASPI aggregations were higher at T2 compared to T1. Sonoclot-analyses showed that activated clotting time (ACT) was unchanged but both clot rate (CR) and platelet function (PF) were higher at T2 compared to T1. VASP decreased from 53 ± 28(T1) to 24 ± 22(T2), (*P <0.001*). The average volume of gastric secretion aspirated before T1 correlated well with VASP (T1), r =0.81 (*P <0.001*). In patients with no platelet inhibition*,* (n =9) similar changes between T1 and T2 were seen as in patients with dual platelet inhibition while VASP was unchanged.

**Conclusions:**

We have demonstrated increased platelet aggregation and strengthened clot formation over time in out of hospital cardiac arrest patients treated with hypothermia. In patients on oral dual platelet inhibition, the effect of ticagrelor was delayed, probably due to slow gastric emptying.

## Introduction

Mild induced hypothermia (MIH) is indicated for comatose survivors of out-of-hospital cardiac arrest (OHCA) to improve neurological outcome [1-3]. However, a recent multicenter study - the target temperature management (TTM) trial [4] in OHCA patients found that a targeted temperature of 33°C did not confer a benefit as compared with a targeted temperature of 36°C and has in some sense challenged current guidelines.

In trauma, hypothermia increases bleeding and worsens outcome [5,6]. Therefore MIH is considered contraindicated in cardiac arrest patients with bleeding and especially intracerebral bleeding [3] and computer tomography (CT) of the brain is often performed prior to MIH. Conventional wisdom holds that hypothermia reduces coagulation, platelet function and impairs primary and secondary haemostasis. Whether this is true also during MIH is still debated [7]. A few animal studies support weakened markers of haemostasis during hypothermia [8-12] while others do not [13-15]. Several reports of studies performed using blood from healthy volunteers, which was incubated at different temperatures, have been published with contradictory results. Some studies show that hypothermia decreases haemostasis [16-21], while others show the opposite [22-26]. Studies including patients treated with MIH after OHCA are more infrequent. In two such studies [27,28] thromboelastography analyses were performed, both studies indicating decreased coagulation with prolonged clot initiation during hypothermia.

Cardiac arrest patients often undergo emergency coronary interventions with stenting, and receive dual antiplatelet therapy, including aspirin and a P2Y_12_ antagonist. The effect of platelet inhibition with the P2Y_12_-antagonist pro-drug clopidogrel may vary secondary to differences in intestinal absorption, variations in liver cytochrome activities, drug interactions, and platelet receptor polymorphisms [29].

Viscoelastic tests such as thromboelastography or Sonoclot do not detect aspirin or P2Y_12_-antagonist effects on haemostasis [30]. With flow cytometry-based vasodilator-stimulated phosphorylated phosphoprotein (VASP) analysis, the effect of P2Y_12_-antagonists can be detected and has been shown to be decreased when clopidogrel is given during MIH [31,32]. To our knowledge there are presently no studies analysing VASP in patients receiving the more potent P2Y_12_-antagonist, ticagrelor, together with aspirin in the OHCA treatment setting. Additionally, cardiac arrest patients develop a systemic inflammatory response syndrome (SIRS) analogous to the changes seen in sepsis, which may be both pro- and antihaemostatic [33-36].

Multiple electrode aggregometry (Multiplate®) is a relatively new tool used to assess adequate patient response to platelet inhibitors [37] and also to evaluate platelet aggregability in sepsis [33,35,36]. The viscoelastic test, Sonoclot has been shown to be superior to thromboelastographic methods for detection of platelet inhibition in hypothermic animals, using glass bead activation [11,15]. It is unknown how haemostasis measured with Multiplate® and Sonoclot is affected after OHCA and MIH in the intensive care setting and the effect of ticagrelor on the VASP analysis is also unexplored. We conducted a prospective observational study in cardiac arrest survivors either with or without ticagrelor and aspirin treatment and assessed haemostasis using Multiplate®, Sonoclot and VASP. We also investigated the relationship between gastric emptying and VASP.

## Methods

This prospective, observational, single-centre study was approved by the regional ethical review board in Lund (registration numbers 411/2004, 223/2008 and 2013/284) and included comatose survivors of OHCA of all origins at the Department of Intensive and Perioperative Care, Skåne University Hospital, Lund, Sweden from February 2012 to October 2013. Informed and written consent was obtained from the next of kin and from all survivors.

Patients were eligible if they had return of spontaneous circulation (ROSC) after non-traumatic OHCA of any origin, were comatose (Glasgow coma scale (GCS) score ≤7) upon admission, and >18 years old. Exclusion criteria were pregnancy, suspicion of intracranial haemorrhage, diagnosed terminal illness, known coagulopathy, and anticoagulant therapy (other than prophylactic dose of low-molecular-weight heparin (LMWH)). Patients were divided into two groups dependent on whether they received dual platelet inhibition or not. All analyses were performed over time within each group; no comparisons were made between the groups.

**Figure 1:**
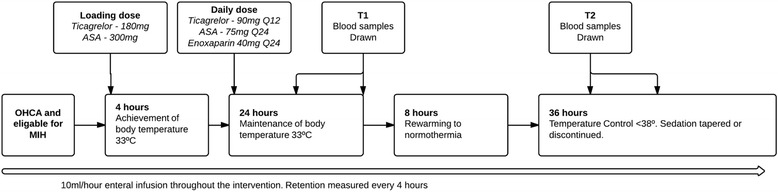
**Flowchart.** Loading and daily doses of ticacrelor and aspirin at the clinicians descretion (n =14). Nine patients did not receive ticagrelor and aspirin. All patients received enoxaparin. T1 blood samples 12 to 24 h after reaching hypothermia. T2 blood samples 16 to 28 hours after reaching normothermia. OHCA, out-of-hospital cardiac arrest; MIH, mild induced hypothermia.

Patients were investigated with CT of the brain to rule out cerebral haemorrhage before inclusion. Coronary angiography and percutaneous coronary intervention (PCI) was performed at the discretion of the treating cardiologist. All patients were treated in accordance with a standardized protocol for MIH. Hypothermia was induced with cold saline (30 ml/kg) and maintained using a cooling device, that is, surface cooling (Arctic Sun®, Medivance®, Louisville, CO, USA) or intravascular cooling (IcyCath®, ZOLL, Sunnyvale, CA, USA) at the discretion of the responsible intensivist. Patients received hypothermia for 24 h at 33 ± 1°C and controlled rewarming at 0.5°C/h. Patients received the following standardized procedures: Foley catheters with incorporated temperature probes, arterial catheters, central venous catheters, and mechanical ventilation after intubation. Patients were sedated with propofol 2 to 4 mg/kg/h and fentanyl 1 to 3 μg/kg/h. Neuromuscular blockade was induced with rocuronium (0.5 mg/kg bolus) if needed, to treat shivering. Enteral nutrition (Isosource® Standard, Nestlé HealthCare Nutrition, New York, NY, USA) was started on arrival at the ICU, at 10 ml/h and continued throughout the study period. Aspiration of gastric secretions from the nasogastric tube was performed every 4 h and enteral nutrition paused if the aspirate exceeded 200 ml. The average aspirated volume was registered. See Gastric secretion below, and flowchart (Figure 1).

### Anticoagulation and platelet inhibition

No patients received thrombolysis. All patients received LMWH, enoxaparin, 40 mg subcutaneously once daily. The clinician decided whether patients should receive dual platelet inhibition with ticagrelor (180 mg loading dose followed by 90 mg twice daily) and aspirin (300 mg loading dose followed by 75 mg daily), normally depending on whether or not emergency PCI was performed. Administration of the dual platelet inhibition was standardized; a loading dose was given 0 to 4 h before target temperature was reached and further maintenance doses were given every 12 (ticagrelor) or 24 (aspirin) h. In the data analysis patients treated with dual platelet inhibition were treated separately from patients not receiving this treatment.

### Blood sampling

Blood was drawn through an arterial catheter using a Safedraw™ PMSET 1DT, (Argon Critical Care Systems, Singapore) and collected in a vacutainer system (BD, Plymouth, UK). The first sampling occasion, time-1 (T1), was during stable hypothermia, that is, 12 to 24 h after reaching target temperature (33°C). The second sampling occasion (T2) was 12 to 28 h after reaching normothermia.

Blood samples were analysed at each time point by conventional coagulation tests (activated partial thromboplastin time (aPTT), prothrombin time, international normalized ratio (PT-INR), platelet count and fibrinogen) along with Multiplate®, Sonoclot, VASP and C-reactive protein (CRP). Blood samples for Multiplate® analysis were collected in 3.0-ml tubes containing recombinant hirudin (Dynabyte GmbH, Munich, Germany). Blood for VASP-analysis was collected in a 4.5-ml tube containing 0.109 M citrate (BD Vacutainer systems, Plymouth, UK). The Sonoclot analysis was performed on native blood collected in a 2-ml syringe (BD, Plymouth, UK).

Conventional haematological tests: PT-INR, aPTT, fibrinogen and platelet count analyses were performed at the accredited hospital laboratory. PT-INR, aPTT and fibrinogen were measured using a Sysmex 5100 analyzer (Siemens Healthcare Diagnostics, Marburg, Germany). The PT-INR assay was performed with the Owren PT reagent (Siemens) calibrated using international normalized ratio (INR) calibrators certified by the Swedish external quality assessment organization EQUALIS AB (Uppsala, Sweden), traceable to World Health Organisation (WHO) RBT/90 standard. The normal value for PT-INR is <1.2. For aPTT, actin FSL and for fibrinogen, Dade Thrombin reagent was used (Siemens). The reference intervals have been established locally for aPTT to 26 to 33 s and for fibrinogen to 2 to 4 g/L.

Platelet count was measured using the Sysmex XN-10 analyzer (Siemens). The locally determined reference range for platelet count is 165 to 387 × 10^9^/L for adult women and 145 to 348 × 10^9^/L for adult men. CRP-was measured using the Cobas 6000/8000 (Roche Diagnostics GmbH, Mannheim, Germany) and a standard procedure; the normal value is <3 mg/L.

VASP was analysed at 37°C using a dual colour flow cytometer assay (PLT VASP/P2Y_12_, BioCytex, Marseille, France), which is specific for P2Y_12_ inhibitors [38]. The ratios between phosphorylated and non-phosphorylated VASP were used to calculate the platelet reactivity index (PRI), which reflects the effect of ticagrelor. According to previous studies, a PRI <50% is regarded as a satisfactory effect of P2Y_12_ inhibitors, a PRI ≥50% is regarded as an unsatisfactory effect of P2Y_12_ inhibitors [39], and PRI ≥70% is considered a normal value for untreated patients.

Multiplate®, multiple electrode aggregometry (Roche, Rotkreuz, Switzerland) was used to measure an agonist-induced platelet aggregation. Hirudin-anticoagulated whole blood was stored at room temperature before analysis within 0.5 to 3.0 h of blood sampling. Both the samples, taken with the patient in stable hypothermia (T1) and in stable normothermia at (T2), were analysed with the Multiplate*®* set first at 33°C in duplicate test cells and then at 37°C in duplicate test cells. The extent of platelet aggregation is measured by resistance (impedance) changes between two electrodes, and then depicted as a graph. The area under the curve (AUC) is the best aggregometry parameter of the Multiplate*®* test. Four test assays were performed; ADPtest (platelet aggregation in response to adenosine-5′-diphosphate), COLtest (platelet aggregation in response to collagen), TRAPtest (platelet aggregation in response to thrombin receptor agonist peptide) and ASPItest (platelet aggregation in response to arachidonic acid).

A Sonoclot Analyzer (Sienco® Inc. Arvada, USA) with a temperature regulated heating or cooling plate was used to study temperature effects on native blood. Both the samples taken with the patient in stable hypothermia (T1) and in stable normothermia at (T2) were analysed within 2 minutes from sampling with the Sonoclot Analyzer set at 33°C and at 37°C. Analyses were performed in duplicate for both temperatures according to the manufacturer’s recommendations. A glass bead test (Sienco® gbACT + ™ Kit), which has been designed to initiate coagulation in a more stable manner than previous tests, was used. The Sonoclot measures the viscoelastic drag-impedance that fibrin and platelets in a whole blood sample impose upon the oscillating Sono-probe. A time-based graph reflects the different steps in the clotting whole blood sample, called the Sonoclot signature. The measured parameters are with defined normal values in parentheses are as follows: 1) ACT (activated clotting time) (100 to 155 s) is the time taken for the first fibrin to form, and is defined as the time it takes for the signature to move 1% upscale from the start of the graph (immersion response) and corresponds to aPTT and traditional ACT tests; 2) clot rate (CR) (9 to 35 units/minute): the rate of increase in the clot impedance due to fibrin formation and polymerization (the slope of the signature after ACT) in % of full scale per minute; 3) platelet function (PF) (>1.5 units) is the point where the squeezing out of trapped serum in the contracting clot (sign of functioning platelets) exceeds the accumulation of clot bulk on the probe and can be described as the time to peak from the immersion response. PF performs better than previous peak amplitude and time to peak parameters, reflecting the gPIIb/IIIa-dependent clot retraction [40].

### Gastric secretion

To estimate the degree of ventricle retention, aspiration of gastric secretions was performed every 4 h. The average aspirated volume during 0 to 24 h after reaching hypothermia (for T1 samples) and during 0 to 28 h after reaching normothermia (for the T2 samples) was registered.

### Statistics

Non-parametric variables (Gaussian distribution not assumed) were summarized using the median with range (minimum to maximum). Parametric variables were summarized using mean ± SD. To reduce the risk of a Type I error due to multiple testing the significance level was set at a *P*-value <0.01. Differences between results were calculated using the two-tailed, Wilcoxon matched-pairs signed test. Correlation coefficients were calculated using Spearman’s rank correlation method. Differences in categorical data were calculated using Fisher’s exact test. All statistical analyses were performed using GraphPad Prism 5, version 5.03; GraphPad Software, La Jolla, CA, USA.

## Results

Twenty-three patients were included, fourteen patients in the group with dual platelet inhibition therapy and nine in the group without platelet inhibition therapy. Demographic data of all subjects are shown in Table 1.

**Table 1:** **Patient demographics**

	**Platelet inhibition** **(n =14 patients)**	**No platelet inhibition ** **(n =9 patients)**
Age, years	66 ± 8	65 ± 13
Male sex, n (%)	10 (71)	8 (89)
Simplified acute physiology score 3	71 ± 16	75 ± 15
Estimated mortality risk, %	54 ± 27	69 ± 24
Bystander cardiopulmonary resuscitation, n (%)	10 (71)	5 (56)
Time to return of spontaneous circulation, minutes	30 (5 to 45)	25 (6 to 37)
30-day mortality, n (%)	8 (58)	5 (56)
Percutaneous coronary intervention, n (%)	12 (86)	0 (0)
**Origin of cardiac arrest**		
Acute myocardial infarction, n (%)	12 (86)	0 (0)
Primary arrhythmia, n (%)	2 (14)	5 (56)
Hypoxia, not hanging (%)	0 (0)	3 (33)
Hanging, n (%)	0 (0)	1 (11)

Results presented as mean ± SD, n (%) or medians (range (minimum to maximum)).

Results from the blood analyses are shown in Table 2. The most important findings are presented below.

**Table 2:** **Blood analyses**

	**With platelet inhibition (n =14 patients)**			**With no platelet inhibition (n =9 patients)**		
	**T1**	**T2**	***P*** **-value**	**T1**	**T2**	***P*** **-value**
**Platelets, 10** ^**9**^ **/L**	189 ± 59	179 ± 64	0.26	206 ± 61	191 ± 68	0.42
**Prothrombin time, international normalized ratio**	1.1 ± 0.1	1.2 ± 0.2	0.03	1.2 ± 0.1	1.4 ± 0.4	0.14
**Activated partial thromboplastin time, s**	34 ± 8	36 ± 11	0.63	34 ± 10	40 ± 17	0.08
**Fibrinogen, g/L**	3.2 ± 0.9	4.9 ± 1.3**	0.002	3.4 ± 1.8	5.1 ± 1.6	0.02
**C-reactive protein, mg/L**	52 ± 38	147 ± 72***	<0.001	73 ± 68	141 ± 28	0.02
**VASP**						
VASP, PRI,%	53 ± 28	24 ± 22***	<0.001	83(75 to 85)	76(50 to 84)	0.38
Samples with adequate effect (PRI <50%), n (%)	7 (50)	12 (86)	0.10	1 (11)	1 (11)	
Samples below normal value (PRI <70%), n (%)	9 (64)	13 (93)	0.16	1 (11)	1 (11)	
**Multiple electrode aggregometry**						
**Adenosine diphosphate-agonist, AUC**	22 (0 to 79)	20 (12 to 43)	0.61	65 (30 to 114)	46 (22 to 145)	0.67
Opposite-analysis temperature	21 (4 to 58)	18 (11 to 52)		62 (24 to 140)	61 (40 to 120)	
**Collagen agonist, AUC**	41 (26 to 62)	56 (25 to 117)***	<0.001	64 (42 to 104)	72 (52 to 147)**	0.0039
Opposite-analysis temperature	42 (27 to 94)	49 (27 to 75)		68 (40 to 123)	74 (60 to 122)	
**Thrombin receptor agonist peptide, AUC**	90 (8 to 116)	99 (82 to 151)**	0.006	83 (40 to 153)	98 (91 to 183)**	0.008
Opposite-analysis temperature	86 (7 to 143)	83 (50 to 107)		89 (44 to 154)	102 (83 to 161)	
**Arachidonic acid-agonist, AUC**	11 (2 to 39)	20 (11 to 62)**	0.003	46 (7 to 130)	66 (18 to 163)**	0.0039
Opposite-analysis temperature	14 (3 to 129)	12 (3 to 28)^a^		50 (9 to 180)	72 (10 to 148)	
**Sonoclot**						
**Activated clotting time, s**	144 (72 to 253)	120 (88 to 191)	0.19	133 (95 to 244)	153 (76 to 213)	0.98
Opposite-analysis temperature	121 (74 to 192)	126 (102 to 226)		120 (79-238)^a^	166 (81 to 223)	
**Clotting rate, units/minute**	25 (15 to 49)	37 (23 to 47)***	<0.001	28 (18 to 64)	41 (24 to 63)	0.44
Opposite-analysis temperature	33 (18 to 50)	32 (15 to 44)^a^		39 (22 to 74)^a^	35 (17 to 46)	
**Platelet function, units**	2.9 (0.4 to 4.4)	3.5 (2.8 to 5.0)***	<0.001	2.9 (0.9 to 4.9)	4.1 (1.8 to 5.2)**	0.0039
Opposite-analysis temperature	4.2 (0.45 to 4.7)^b^	3.2 (1.3 to 4.2)^a^		3.9 (1.9 to 5.0)	3.4 (2.7 to 5.1)	

Results presented as mean ± SD, median (range (minimum to maximum), or number (%). T1, blood sampling 12 to 24 h after reaching 33°C); T2 blood sampling 16 to 28 h after reaching normothermia. Multiple electrode aggregometry, (Multiplate®) and Sonoclot analyses set on the patient’s body temperature at the sampling occasion and at Opposite-analysis temperature (that is, 37°C if the patient’s body temperature was 33°C and vice versa). Differences between laboratory results for T2 versus T1 (*P*-values) were calculated using two-tailed, paired *t*-test for means, two-tailed paired Wilcoxon matched pairs signed test for medians and Fisher’s exact test for categorical variables. **P <0.01. ***P <0.001. ^a^ *P* <0.01 compared to the other analysed temperature. ^b^ *P* <0.001 compared to the other analysed temperature on the same sampling occasion. VASP, vasodilator-stimulated phosphorylated phosphoprotein; PRI, platelet reactivity index; AUC: area under the curve.

**Figure 2:**
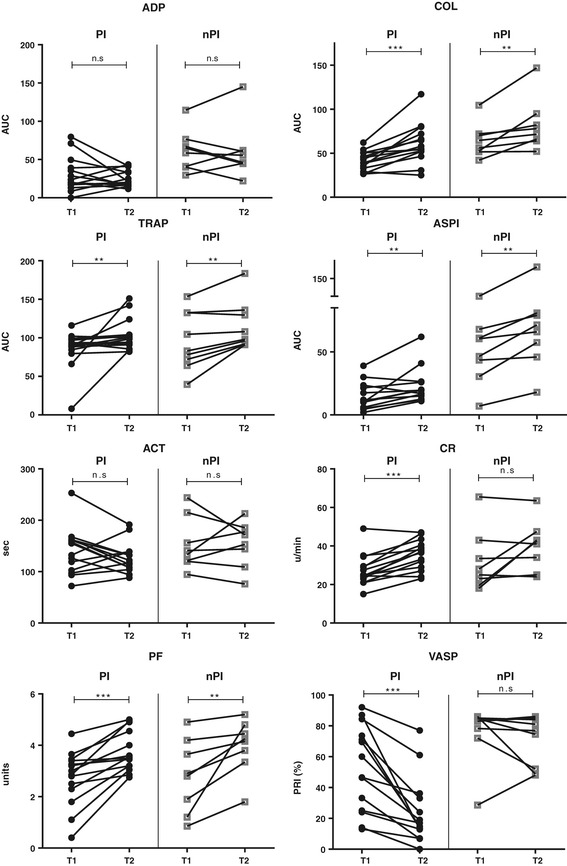
**Results from blood analyses for individual patients.** Multiplate® and Sonoclot instruments set on the *in-vivo* temperature. PI, patients with dual platelet inhibition (n =14); nPI, patients with no platelet inhibition (n =9); T1, blood sampling 12 to 24 h after reaching 33°C body temperature; T2, blood sampling 16 to 28 h after reaching normothermia. Multiple electrode aggregometry, Multiplate®: ADP: adenosine diphosphate-agonist; COL, collagen-agonist; TRAP, thrombin-agonist; ASPI, arachidonic-acid agonist. Sonoclot analyses: ACT, activated clotting time; CR, clotting rate; PF, platelet function. AUC, area under curve; **P <0.01; ***P <0.001.

Patients with dual platelet inhibition: platelet count, PT-INR and aPTT were all unchanged between T1 and T2. Fibrinogen increased from 3.2 ± 0.9 (T1) to 4.9 ± 1.3 (T2), (*P* =0.002) and CRP increased from 52 ± 38 (T1) to 147 ± 72 (T2) (*P* <0.001). VASP decreased from 53 ± 28 (T1) to 24 ± 22 (T2), (*P* <0.001). Multiplate®-analyses, with the analyses temperature set on the *in vivo* temperature, showed no changes in ADP-stimulated platelets. COL, TRAP and ASPI tests were all increased at T2 compared to T1 (Table 2 and Figure 2). In the Sonoclot-analyses ACT was unchanged but both CR and PF was increased at T2 compared to T1 (Table 2 and Figure 2).

Patients with no platelet inhibition: platelet count, PT-INR, aPTT, and VASP did not change significantly. Multiplate®-analyses were performed with the analyses temperature set on the patient’s body temperature. ADP and TRAP tests were not changed significantly. COL and ASPI tests increased at T2 compared to T1 (Table 2 and Figure 2). In the Sonoclot®-analyses ACT and CR were unchanged but PF was increased in T2 compared to T1 (Table 2 and Figure 2).

**Figure 3:**
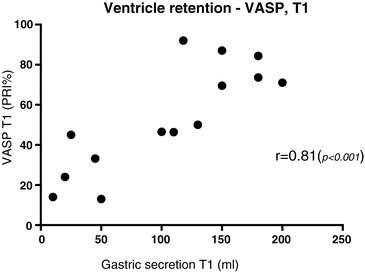
**Correlation between aspirated gastric secretion and vasodilator-stimulated phosphorylated phosphoprotein (VASP)** **.** Patients with dual platelet inhibition (n =14). Gastric secretion T1, median volume of gastric secretion aspirated from nasogastric tube, 0 to 24 h after reaching 33°C body temperature; VASP T1, 12 to 24 h after reaching 33°C body temperature; PRI, platelet reactivity index.

Correlation aspirated gastric secretion - VASP: the median volume of gastric secretion aspirated in patients with dual platelet inhibition was 105 (10 to 200) ml during T1 and 65 (10 to 200) ml during T2 (not significant). The volume of gastric secretion aspirated during T1 correlated well with VASP (T1), *r* =0.81 (*P* <0.001) (Figure 3). This correlation was not detected at T2.

## Discussion

In this prospective observational study on OHCA patients treated with MIH we have demonstrated an increase in Multiplate®-assays COL, TRAP, ASPI and Sonoclot assays CR and PF between the stable hypothermic and the stable normothermic state, indicating increased platelet aggregation and strengthened clot formation.

This observational study did not include a normothermic control group, thus the cause for the increased platelet aggregability and viscoelastic clot formation between stable hypothermia (T1) and stable normothermia (T2) is not known but two possible explanations will be discussed. Hypothermia has been shown to decrease haemostasis measured with viscoelastic methods [11,18,19,28] and with platelet function testing [16,17,21]. Thus, the lower temperature at T1 could be responsible for the decreased values as compared to T2. In addition, post-resuscitation stress responses after cardiac arrest mimic the immunologic and coagulation disorders observed in severe sepsis [41]. Presumably the low-flow state during cardiac arrest, followed by a reperfusion injury after return of spontaneous circulation is responsible for a SIRS reaction that may cause an activation of the coagulation system [36,42]. This can be compared to studies by Adamzik *et al*. [33] and Brenner *et al*. [35] that showed reduced platelet aggregation, measured with Multiplate®, in patients with severe sepsis. In our study CRP and fibrinogen increased between the sampling occasions (Table 2) in all patients indicating a significant SIRS with an acute phase reaction 2 to 3 days after OHCA, although this did not reach statistical significance in the group without platelet inhibition.

The effect of ticagrelor depends on intestinal absorption to the systemic circulation. It is well-known that hypothermia, opioids and acute critical illness reduce gastrointestinal motility [43]. In the present study 50% of the patients with dual platelet inhibition did not reach the target VASP PRI <50% at the first sampling occasion (T1), thus placing them at risk for thromboembolic events. This is in agreement with previous findings of impaired bioavailability of clopidogrel in critically ill patients [44] and comparable with the results from Bjelland *et al*. [31] and Ibrahim *et al*. [32], which demonstrated high rates of non-responders in clopidogrel-treated patients (100% and 83%, respectively) as well as in ticagrelor-treated patients (61%) [45] in MIH after cardiac arrest. In the present study we also demonstrated significant correlation between gastric emptying and VASP during hypothermia (Figure 3), indicating that gastric emptying is responsible for the limited ticagrelor effect observed at T1. These findings underline the importance of stimulating motility of the gastro-intestinal tract as soon as possible in the cardiac arrest patient who is dependent on absorption of oral P2Y_12_ inhibitors to limit the risk of stent thrombosis. This is also demonstrated in a recent observational study by Joffe *et al*. [46], including patients with acute myocardial infarction treated with coronary stents. In the study by Joffe *et al*. cardiac arrest patients did not receive P2Y_12_ inhibitors and had a much higher incidence of stent thrombosis (10.9%) than patients in the control group (2.0%), who were treated with P2Y_12_ inhibitors but had not had previous cardiac arrests.

VASP analysis is specifically designed to monitor only the P2Y_12_ platelet inhibitory effect on platelets [38] and as expected, only patients on P2Y_12_ platelet inhibitory medication actually exhibited VASP changes. Multiplate® and Sonoclot tests on the other hand, are not as specific as VASP analysis and are sensitive to many pro- and anti-haemostatic variables [24,25,33,35,47], not detectable with VASP. Furthermore, Multiplate®-assays (except ADPassay) and Sonoclot assays cannot detect moderate P2Y_12_inhibition [30]. These characteristics of the different analyses explain why Multiplate®-assays, COL, TRAP, ASPI and Sonoclot assays, CR and PF, in our study show strengthened platelet function and coagulation over time, and on the other hand, P2Y_12_-sensitive VASP analysis show weakened platelet function in ticagrelor treated patients.

We performed all Multiplate® and Sonoclot analyses with the instruments set both at the patient’s actual body temperature (the *in vivo* temperature) and at the opposite temperature, that is, 33°C or 37°C. This was not performed to determine whether the rise in body temperature or the SIRS is responsible for the increased values in normothermia, but rather to identify how the temperature of the instrument affects the results. We found that ASPItest in the Multiplate® analyses and multiple assays in the Sonoclot analyses indicated weaker values when normothermic samples were cooled and stronger values when hypothermic samples were warmed (Table 2). This is in agreement with results from Shimokawa *et al*. [11] that showed the importance of performing hemostatic measurements with the Sonoclot and thromboelastography at the actual *in vivo* (hypothermic) temperature and not only at the traditional 37°C setting. The PF platelet parameter used in the present study better reflects clot contraction than the parameters used by Shimokawa [40].

Other limitations of the present study include that platelet function assay was done under minimal shear, so that platelet function under flow, which is influenced by haematocrit and von Willebrand Factor, could not be assessed [48]. Furthermore, this study did not explore the causes for the increased platelet aggregability and the viscoelastic clot formation. We welcome a randomized controlled trial to explore whether hypothermia, the SIRS reaction, an unknown factor or a combination of factors are responsible for the observed changes.

## Conclusions

We have demonstrated increased platelet aggregation and strengthened clot formation over time in out-of-hospital cardiac arrest patients treated with hypothermia. In patients on oral dual platelet inhibition, the effect of ticagrelor was delayed, probably due to slow gastric emptying.

## Key messages

- Platelet aggregation and clot formation demonstrated with Multiplate® and Sonoclot are strengthened over time in out-of-hospital cardiac arrest patients treated with hypothermia
- Fifty percent of the patients on oral ticagrelor did not reach the target VASP PRI <50% at the first sampling occasion 12 to 24 hours after reaching hypothermia, thus placing them at risk for thromboembolic events
- The effect of ticagrelor was delayed in survivors of cardiac arrest, probably due to slow gastric emptying

## Abbreviations

ACT: activated clotting time
ADP: adenosine-5′-diphosphate agonist
aPTT: activated partial thromboplastin time
ASPI: arachidonic acid agonist
COL: collagen agonist
CR: clot rate
CRP: C-reactive protein
GCS: Glasgow coma scale
LMWH: low-molecular-weight heparin
MIH: mild induced hypothermia
nPI: non-platelet inhibition
OHCA: out-of-hospital cardiac arrest
PCI: percutaneous coronary intervention
PF: platelet function
PI: platelet inhibition
PRI: platelet reactivity index
PT-INR: prothrombin time international normalized ratio
ROSC: return of spontaneous circulation
SAPS3: simplified acute physiology score 3
SIRS: systemic inflammatory response syndrome
TRAP: thrombin receptor agonist peptide
VASP: vasodilator-stimulated phosphoprotein
